# Identifying the Associations of Nightly Fasting Duration and Meal Timing with Type 2 Diabetes Mellitus Using Data from the 2016–2020 Korea National Health and Nutrition Survey

**DOI:** 10.3390/nu15061385

**Published:** 2023-03-13

**Authors:** Junkyung Kwak, Kyeong-A Jang, Haeng-Ran Kim, Min-Sook Kang, Kyung Won Lee, Dayeon Shin

**Affiliations:** 1 Department of Food and Nutrition, Inha University, Incheon 22212, Republic of Korea; 2 Department of Agro-Food Resources, National Institute of Agricultural Sciences, Rural Development Administration, Wanju 55365, Republic of Korea; 3 Department of Home Economics Education, Korea National University of Education, Cheongju 28173, Republic of Korea

**Keywords:** nightly fasting duration, meal timing, type 2 diabetes mellitus, Korea National Health and Nutrition Survey, Korean adults

## Abstract

Nightly fasting duration and meal timing are associated with metabolic disorders. This study aimed to investigate the relationships of nightly fasting duration and meal timing with type 2 diabetes mellitus (T2DM) using data from the 2016–2020 Korea National Health and Nutrition Survey. A total of 22,685 adults ≥ 19 years were included in this study. Nightly fasting duration was calculated by subtracting the interval between the day’s first and last meal eating times from 24 h. The meal timing were analyzed using various parameters, including the times of the first and last eating episodes and the percentage of energy intake during the morning (05:00 to 9:00 a.m.), evening (06:00 to 09:00 p.m.), and night (after 09:00 p.m.). Men who fasted nightly for ≥ 12 h had lower odds of T2DM (odds ratio (OR): 0.86; 95% confidence interval (CI): 0.75–0.99) than those who fasted for < 12 h. Individuals who had their last meal after 09:00 p.m. had higher odds of T2DM (OR: 1.19, 95% CI: 1.03–1.38, men; OR: 1.19, 95% CI: 1.01–1.40, women). Additionally, the percentage of energy intake during the evening was associated with increased odds of T2DM (OR: 1.41, 95% CI: 1.08–1.84, men; OR: 1.32, 95% CI: 1.02–1.70, women). These findings emphasize the importance of nightly fasting duration and meal timing in modulating the risk of T2DM among Korean adults.

## 1. Introduction

The prevalence of type 2 diabetes mellitus (T2DM) is continuously increasing due to diet changes, increased stress, and decreased physical activity. In Korea, the prevalence of chronic diseases, including T2DM, increased in 2020 [1]. According to 2020 statistics, one out of ten (10.7%) adults aged ≥ 19 years had T2DM and the prevalence of T2DM was 14.8%, particularly among the low-income groups [1]. Moreover, since T2DM is accompanied by various complications, such as chronic kidney disease, cardiovascular disease, and stroke, its prevention and treatment are essential [2]. Diet and lifestyle changes are important for T2DM prevention and management [3]. In a US clinical trial of 3234 adults, lifestyle improvements, including a healthy, low-calorie, low-fat diet and moderate-intensity physical activity, reduced the incidence of T2DM by 58% [4]. Additionally, a healthy lifestyle approach was more effective in reducing the incidence of T2DM than a pharmacological method with metformin administration [4].

Fasting duration has received considerable attention as one of the dietary factors related to T2DM. Time-restricted eating, a type of fasting, usually begins in the evening or at night and requires fasting for 12–18 h [5]. Intermittent fasting, which extends the night fasting period to more than 8 h [6], has many advantages for improving T2DM-related metabolic markers [7,8]. Intermittent fasting increases plasma insulin by increasing autophagy in pancreatic islets, which decreases blood glucose levels and improves glucose tolerance [9]. A previous intervention study with 19 participants showed that fasting for 10 h per day for 12 weeks improved fasting blood glucose, fasting insulin, and glycated hemoglobin (HbA1c) levels [10]. Previous randomized controlled trials (RCTs) demonstrated that daily 16 h fasting increased glucose tolerance [11], decreased 24 h glucose levels, blood glucose excursions [12], and homeostatic model assessment for insulin resistance (HOMA-IR) [13]. In another RCT, daily 21 h fasting for 8 weeks decreased fasting blood glucose levels in 13 healthy middle-aged adults [14]. The 14 h fasting was associated with increased glucose tolerance and decreased blood glucose, HOMA-IR, and HbA1c [10,11,12,13,14].

During overnight fasting, prolonged fasting ketosis, in which fatty acids are converted into ketones after glucose exhaustion, may contribute to improved regulation of glucose metabolism [15,16]. The secretion of blood glucose and insulin has a circadian rhythm, and glucose homeostasis is controlled by time. Since glucose metabolism is affected by circadian rhythms, glucose tolerance usually peaks during daylight when food consumption begins and decreases during darkness when fasting occurs [17]. When the same oral glucose solution was provided, plasma glucose levels were found to be higher during dinner than during breakfast in humans [18]. Similar findings were also observed in rodents whose main activity period was at night and who had lower glucose tolerance during the day than at night [19]. Although studies on the association between nightly fasting duration and T2DM are limited, they have shown that nightly fasting duration is associated with the risk of T2DM, metabolic syndrome, and obesity [20,21]. According to the 2005–2016 National Health and Nutrition Examination Survey, a long nightly fasting duration was associated with increased insulin (β: 0.29, *p* < 0.01) and C-reactive protein (β: 0.03, *p* = 0.02) levels and decreased high-density lipoprotein (HDL)-cholesterol (β: −0.10, *p* = 0.03) levels in US adults [20]. Another cross-sectional study found that nightly fasting duration was negatively associated with the odds of elevated triglycerides (odds ratios (OR): 0.73, 95% confidence interval (CI): 0.55–0.98) and metabolic syndrome (OR: 0.74, 95% CI: 0.55–0.99), but not associated with elevated fasting blood glucose (OR: 0.85, 95% CI: 0.64–1.12) in Iranian adults [21]. In a cross-over study, nightly fasting duration was negatively associated with body weight in healthy young men [22].

In addition to fasting duration, meal timing is an important factor that influences the interaction between the circadian clock and metabolism in the body [23]. Metabolic disorders can be affected not only by the quality and quantity of meals but also by the times of food intake [24]. Many studies have investigated the effect of meal timing on diabetes-related parameters [25,26,27,28,29,30,31]. High-energy intake from breakfast with a low-energy dinner is negatively associated with daily postprandial hyperglycemia in patients with diabetes [25]. Due to circadian regulation, meals consumed in the afternoon and evening have been shown to result in higher glucose tolerance and lower insulin sensitivity and β-cell responsiveness compared to breakfast [26]. In addition, skipping breakfast increases the glycemic response to lunch and dinner [25], and postprandial glucose levels are higher during dinner than during breakfast [27]. Late-night meals cause postprandial hyperglycemia in patients with diabetes [28]. In addition, restricting meals at night has a preventive effect on metabolic disorders, including glucose intolerance, which can be caused by a high-fat diet [29]. Changes in meal timing due to shifts in circadian rhythms for various reasons, such as jet lag and shift work, have been associated with increased blood glucose levels and glucose intolerance [26,30].

Most studies have been conducted on intermittent fasting and fasting during the day, and studies examining the relationships between nightly fasting duration and the risk of T2DM in Korean adults are limited [25,26,27,28,29,30]. Thus, this study aimed to investigate the associations of nightly fasting duration and meal timing with the odds of T2DM based on a representative sample of Korean adults using data from the 2016–2020 Korea National Health and Nutrition Examination Survey (KNHANES).

## 2. Materials and Methods

### 2.1. Data Source and Study Participants

**Figure 1:**
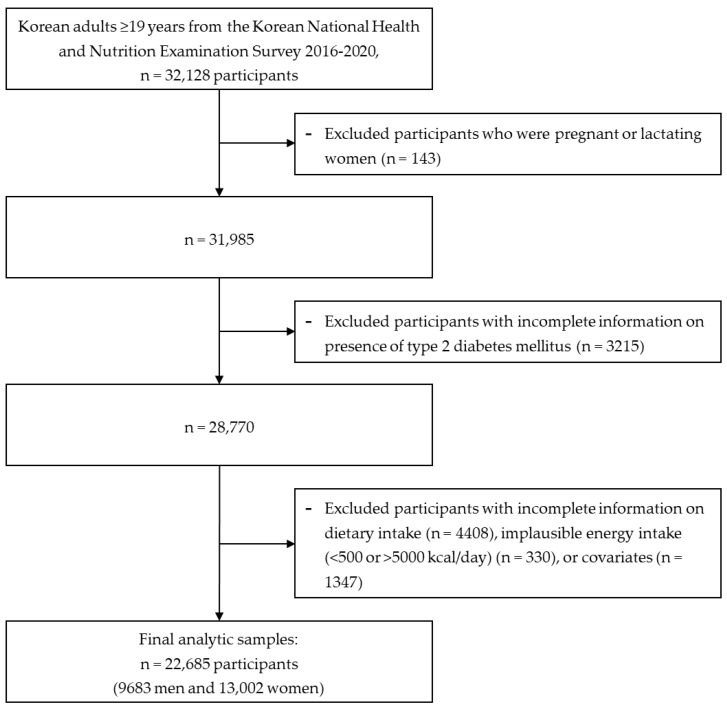
Flow diagram of the study population.

Data from the 2016–2020 KNHANES were used to investigate the associations between nightly fasting duration and meal timing and the risk of T2DM (Figure 1). Using a structured questionnaire, information about health behaviors, such as smoking and drinking status, physical activity, mental health, and the type and amount of food consumed, as well as a health examination, including blood and urine tests, were collected.

Using the data of 32,128 study participants, those who were pregnant and lactating (n = 143), had no T2DM information (n = 3215), had incomplete dietary data (n = 4408), had an implausible energy intake (<500 kcal/day or >5000 kcal/day) (n = 330), or had insufficient information on covariates, such as educational level, household income level, household type, alcohol consumption, smoking status, regular physical activity, and body mass index (BMI) (n = 1347), were excluded. Finally, 22,685 Korean adults (9683 men and 13,002 women) were included to analyze the relationships between nightly fasting duration, meal timing, and the odds of T2DM. All methods and protocols were conducted in accordance with the relevant institutional guidelines and regulations, and all participants provided written informed consent. This study was reviewed and approved by the Institutional Review Board of the Korea National University of Education (IRB no. KNUE-202208-BM-0322-01).

### 2.2. Calculation of Nightly Fasting Duration and Meal Timing

Nightly fasting duration and meal timing were defined using dietary data collected by a 24 h recall survey of nutrition survey items. The 24 h recall data provided time-of-day information for all consumed foods and beverages. The nightly fasting duration was calculated by subtracting the interval between the first and last meal of the day from 24 h. Three types of nightly fasting durations were included in the analysis: categorical (quartiles and median) and continuous. Meal timing were analyzed using various parameters, including the first and last meal timing and the percentage of energy intake during the morning (05:00 to 9:00 a.m.), evening (06:00 to 09:00 p.m.), and night (after 09:00 p.m.).

### 2.3. Assessment of Type 2 Diabetes Mellitus

This study used the fasting glucose and HbA1c values obtained from blood samples after fasting for 8 h. The use of oral anti-diabetic medications or insulin and a physician’s diagnosis were recorded as yes or no. T2DM was defined as a fasting glucose level ≥ 126 mg/dL, the use of oral antidiabetic medications or insulin, a physician’s diagnosis, or HbA1c ≥ 6.5% [31].

### 2.4. Statistical Analyses

All statistical analyses were performed using the SAS software (version 9.4; SAS Institute, Cary, NC, USA). Statistical significance was set at a *p* value of <0.05. The participants’ general characteristics, nutrient and meal intake, and dietary behaviors according to the nightly fasting duration were compared using chi-square tests for the categorical variables and multiple linear regressions for the continuous variables, which are presented as numbers (weighted percentages) or means ± standard errors. Multivariable logistic regression models were used to calculate the odds ratios (ORs) and 95% confidence intervals (CIs) of T2DM according to the nightly fasting duration (quartiles, medians, and continuous) and mealtime variables (first and last meal timing and the percentage energy intake during the morning, evening, and night). All the analyses were stratified by sex. The age-adjusted model was adjusted for age, whereas the multivariable-adjusted model was adjusted for various covariates, including age (continuous), education level (elementary school, middle school, high school, or college), household income (lower-middle, middle, upper-middle, or high), household type (single or multi persons), alcohol consumption (none, moderate, or high), smoking status (never, past, or current), regular physical activity (yes or no), and BMI (kg/m^2^, continuous).

## 3. Results

### 3.1. General Characteristics of Study Participants According to Nightly Fasting Duration

Table 1 shows the general characteristics of the nightly fasting duration according to the quartile of nightly fasting duration. The nightly fasting duration was significantly associated with age, education level, household income, alcohol consumption, and smoking status among both men and women (all *p* < 0.05). According to the nightly fasting duration, significant differences were found in household type and regular physical activity among men, and BMI among women (all *p* < 0.05). Regardless of sex, individuals in the highest quartile of nightly fasting duration were more likely to be younger, educated, have lower household incomes, live in single-person households (only in men), and have a lower BMI (only in women), and less likely to be current smokers and have higher alcohol consumption, regular physical activity (only in men) compared to those in the lowest quartile (all *p* < 0.05).

### 3.2. Nutrient and Meal Intake and Dietary Behaviors of Study Participants According to Nightly Fasting Duration

Table 2 shows the nutrient and meal intake of the study participants based on the quartiles of nightly fasting duration. Participants in the highest quartile of nightly fasting duration had lower total energy intake, percentage of energy from carbohydrates, dietary fiber, and percentage of energy from breakfast and snacks, but a higher percentage of energy from protein, fats, main meals, lunch, and dinner compared with those in the lowest quartile (all *p* < 0.01) in both men and women.

Table 3 shows the dietary behaviors of the study participants according to the quartiles of nightly fasting duration. In both men and women, individuals with the longest nightly fasting duration tended to have lower frequencies of total eating, main meal, and snack episodes. They also had a longer nightly fasting duration, later timing of the first eating episode, and earlier timing of the last eating episode compared to those with the shortest duration (all *p* < 0.01). Men and women in the highest quartile also had lower energy intake from the morning and night eating episodes, and foods consumed away from home, and higher energy intake in the evening compared to those in the lowest quartile of nightly fasting duration (all *p* < 0.01).

### 3.3. Relationship between Nightly Fasting Duration and Risk of Type 2 Diabetes Mellitus 

Table 4 shows the odds of T2DM according to the quartiles, medians, and continuous types of nightly fasting duration. The median value of nightly fasting duration was 12.0 h for men and 12.7 h for women. In the fully adjusted model, men in the highest nightly fasting duration quartile had decreased odds of T2DM compared to men in the lowest quartile (multivariable-adjusted OR: 0.75, 95% CI: 0.61–0.91). However, there was no significant association between nightly fasting duration and the odds of T2DM in men, regardless of whether nightly fasting duration was included as a median or continuous variable in the model. In women, those in the highest quartile of nightly fasting duration had decreased odds of T2DM compared to those in the lowest quartile (multivariable-adjusted OR: 0.77, 95% CI: 0.62–0.95). In addition, every 1 h increase in the nightly fasting duration was significantly associated with decreased odds of T2DM in women (multivariable-adjusted OR: 0.97, 95% CI: 0.94–0.99). However, on a median value basis, there was no significant association between nightly fasting duration and the odds of T2DM in women.

### 3.4. Relationship between Meal Timing and Type 2 Diabetes Mellitus Risk

Table 5 presents the associations between meal timing and the odds of T2DM. Individuals who had their last meal after 09:00 p.m. had increased odds of T2DM compared to those who had their last meal before 09:00 p.m. in both men and women (multivariable-adjusted OR: 1.18, 95% CI: 1.02–1.37 for men; multivariable-adjusted OR: 1.20, 95% CI: 1.02–1.40 for women). Compared to those who had no energy intake in the evening, individuals who had ≥ 40% energy intake in the evening had increased odds of T2DM in both sexes (multivariable-adjusted OR: 1.40, 95% CI: 1.08–1.83 for men; multivariable-adjusted OR: 1.32, 95% CI: 1.02–1.70 for women). The percentage of energy intake at night showed a significant association with the odds of T2DM only in women. Women who had ≥ 25% energy intake at night had increased odds of T2DM compared to those who had no energy intake at night (multivariable-adjusted OR: 1.61, 95% CI: 1.13–2.30). However, the time of the first eating episode and percentage of energy intake in the morning had no significant associations with T2DM in Korean adults.

## 4. Discussion

This nationwide cross-sectional study found that nightly fasting duration and meal timing were associated with the risk of T2DM in Korean adults after adjusting for various confounding factors based on data from the 2016–2020 KNHANES. In this study, nightly fasting duration was negatively associated with the odds of T2DM in both sexes, and every 1 h increase in the nightly fasting duration was also negatively associated with T2DM in women. Consistent with our findings, time-restricted eating with an extended nightly fasting duration decreased blood glucose, HOMA-IR, and HbA1c and improved glucose tolerance [10,11,12,13,14]. In addition, an increased nightly fasting duration was associated with decreased blood glucose, HOMA-IR, and increased insulin levels [20,21].

In addition to the nightly fasting duration, to examine whether meal timing were associated with the odds of T2DM, the relationships between the first and last meal timing, as well as the percentage of energy intake in the morning, evening, and night, and T2DM were also analyzed. In both men and women, having the last eating episode after 9:00 p.m. was positively associated with the odds of T2DM. However, no significant association between the time of the first eating episode and T2DM was reported. A cross-sectional study with a nationally representative sample of US adults found that a later time of the first meal increased levels of insulin, blood glucose, and HbA1c, and a later time of the last meal increased levels of HbA1c [20]. Consequently, the earlier the timing of the last eating episode, the more beneficial for T2DM-related parameters such as blood glucose, insulin, and HbA1c [20], suggesting that eating the last meal before 9:00 p.m. and longer nightly fasting duration may help prevent T2DM.

Meal timing are related to the circadian clock [32], which regulates glucose metabolism by controlling metabolic enzymes, hormones, and transport systems [17]. Changes in modern lifestyle, such as shift work, jet lag, a lack of sleep, late eating times, and late chronotypes, lead to circadian disturbances [33,34,35,36,37,38,39], thereby causing glucose dysregulation in healthy adults [40]. In this study, we investigated the association between energy intake in the morning, evening, and night and T2DM. The present study reported that there was no significant association between the percentage of energy intake in the morning and T2DM in either men or women. Contrary to our findings, previous studies have reported a negative association between breakfast and T2DM [41,42]. Skipping breakfast contributes to impaired blood glucose control and an increase in blood glucose levels during lunch [41]. Additionally, skipping breakfast is related to a decrease in insulin and an increase in ghrelin levels, which causes overeating and chronic diseases, including T2DM [42]. The discrepancy between the findings of the present study and those of previous studies can be explained by the difference in the definitions of breakfast. Although previous studies focused on whether or not to eat breakfast, we considered the percentage of energy consumed in the morning.

The results of this study showed that consuming ≥ 40% energy in the evening was positively associated with a higher odds of T2DM in both men and women, and consuming ≥ 25% energy at night was positively associated with a higher odds of T2DM in women. Consistent with our findings, previous studies also reported a positive association between evening and nighttime energy intake and T2DM [28,43,44,45,46,47,48]. Many T2DM patients tend to eat emotionally at night [43], and having a high-energy dinner has been reported to double the incidence of T2DM [44]. Compared to daytime meals, nighttime meals increase blood glucose levels and HbA1c and have been associated with two or more diabetic complications by disrupting the 24 h circadian rhythms [43,45,46,47]. Consuming habitual late-night meals may also lead to hyperglycemia due to factors such as skipping breakfast and eating alone [48]. Previous studies have shown that having dinner at 9:00 p.m. contributes to increased blood glucose compared to having dinner at 6:00 p.m [28]. However, sharing one meal twice is beneficial for preventing T2DM, especially when it is inevitable to have a late dinner for various reasons, such as shift work or jet lag [28]. Since eating after 9:00 p.m. is significantly related to the risk of T2DM, increasing the duration of nightly fasting and having an early last meal may help prevent T2DM.

This study has several limitations. First, since this study has a cross-sectional design, the causal relationship of nightly fasting duration and meal timing with T2DM cannot be established. Second, based on the 24 h recall methods, we calculated the first and last meal timing of the day and then the nightly fasting duration was estimated. Therefore, there may be a difference between the actual and estimated nightly fasting durations. Third, because this study was conducted on Korean adults, it is difficult to generalize the results to other populations. Finally, although diet quality is very important for individuals with T2DM [49], meal quality was not considered because this study focused on nightly fasting duration and meal timing. Despite these limitations, the strength of this study is that it is the first to comprehensively investigate the associations between nightly fasting duration and meal timing, and T2DM in Korean adults by adjusting for various confounding variables. Additional prospective studies are needed to investigate the causal relationships of nightly fasting duration and meal timing with T2DM. Furthermore, it is necessary to consider dietary quality and indexes such as the glycemic index, which affects blood glucose levels.

## 5. Conclusions

We demonstrated the associations between nightly fasting duration and meal timing and T2DM in Korean adults. Our findings emphasize the importance of nightly fasting duration and meal timing and suggest that time-related healthy eating patterns, particularly an increased nightly fasting duration, the early timing of the last eating episode, and reduced energy intake in the evening and at night, might be beneficial for T2DM prevention and treatment.

## Figures and Tables

**Table 1:** General characteristics of study participants according to quartiles of nightly fasting duration in Korean adults.

	Men (n = 9683)					Women (n = 13,002)				
	Nightly Fasting Duration				*p* Value ^(1)^	Nightly Fasting Duration				*p* Value ^(1)^
	Q1 (Lowest)	Q2	Q3	Q4 (Highest)		Q1 (Lowest)	Q2	Q3	Q4 (Highest)	
	(n = 2680)	(n = 1954)	(n = 2728)	(n = 2321)		(n = 3524)	(n = 3073)	(n = 3627)	(n = 2778)	
Age, years	45.4 ± 0.4	49.6 ± 0.4	49.9 ± 0.4	41.3 ± 0.4	<0.0001	48.5 ± 0.3	50.6 ± 0.3	50.1 ± 0.4	42.0 ± 0.4	<0.0001
Education level					<0.0001					<0.0001
≤Elementary	228 (4.9)	236 (7.9)	403 (9.7)	200 (8.6)		639 (13.3)	696 (16.6)	930 (19.0)	418 (10.4)	
Middle	242 (6.5)	235 (8.6)	314 (8.3)	188 (8.1)		397 (9.4)	394 (11.3)	414 (9.6)	222 (6.2)	
High school	802 (29.4)	555 (27.7)	737 (26.4)	576 (24.8)		1074 (31.3)	787 (27.5)	882 (25.8)	720 (25.4)	
≥College	1408 (59.2)	928 (55.9)	1274 (55.6)	1357 (58.5)		1414 (46.0)	1196 (44.6)	1401 (45.6)	1418 (57.9)	
Household income					<0.0001					<0.0001
Low	249 (7.3)	272 (10.5)	494 (13.3)	368 (12.0)		452 (10.1)	503 (12.8)	718 (15.2)	429 (12.3)	
Lower-middle	454 (14.3)	371 (17.3)	535 (17.2)	401 (15.7)		658 (17.4)	599 (17.6)	701 (17.5)	503 (17.2)	
Middle	588 (22.5)	386 (20.7)	516 (19.6)	464 (20.0)		720 (21.1)	603 (19.5)	745 (21.7)	594 (22.7)	
Upper-middle	688 (27.3)	441 (23.4)	546 (22.4)	524 (25.1)		785 (24.1)	684 (24.7)	724 (22.1)	656 (24.5)	
High	701 (28.6)	484 (28.1)	637 (27.5)	564 (27.1)		909 (27.3)	684 (25.4)	739 (23.5)	596 (23.3)	
Household type					0.0002					0.1042
Single-person	299 (9.7)	203 (9.0)	309 (10.1)	320 (13.2)		410 (9.0)	403 (9.3)	530 (10.5)	385 (10.4)	
Multi-person	2381 (90.3)	1751 (91.0)	2419 (89.9)	2001 (86.8)		3114 (91.0)	2670 (90.7)	3097 (89.5)	2393 (89.6)	
Alcohol consumption					0.0057					<0.0001
None	769 (27.1)	591 (29.2)	912 (31.6)	703 (28.2)		2002 (52.8)	1901 (58.1)	2255 (57.8)	1511 (50.4)	
Moderate	946 (37.5)	704 (38.3)	885 (35.1)	844 (39.9)		1055 (33.0)	856 (30.1)	1019 (31.6)	921 (36.8)	
High	965 (35.4)	659 (32.4)	931 (33.3)	774 (31.9)		467 (14.1)	316 (11.8)	353 (10.5)	346 (12.7)	
Smoking status					<0.0001					<0.0001
Never	576 (23.9)	464 (25.5)	668 (26.2)	674 (32.8)		3080 (85.7)	2799 (90.3)	3294 (89.7)	2406 (85.2)	
Past	1077 (37.6)	926 (44.5)	1323 (44.2)	901 (33.0)		233 (7.0)	151 (5.0)	187 (5.5)	227 (8.7)	
Current	1027 (38.5)	564 (30.1)	737 (29.6)	746 (34.3)		211 (7.3)	123 (4.6)	146 (4.8)	145 (6.0)	
BMI, kg/m^2^	24.7 ± 0.1	24.6 ± 0.1	24.5 ± 0.1	24.8 ± 0.1	0.0680	23.3 ± 0.1	23.3 ± 0.1	23.4 ± 0.1	23.1 ± 0.1	0.0273
Physical activity					0.0017					0.1104
Yes	1377 (48.3)	1078 (52.1)	1539 (52.3)	1172 (47.2)		2050 (55.4)	1885 (58.7)	2136 (56.1)	1619 (55.6)	
No	1303 (51.7)	876 (47.9)	1189 (47.7)	1149 (52.8)		1474 (44.6)	1188 (41.3)	1491 (44.0)	1159 (44.4)	

KNHANES, Korea National Health and Nutrition Examination Survey; Q, quartile; BMI, body mass index. Data are presented as means ± standard errors (SEs) or numbers (weighted %). ^(1)^ *p* values were calculated using the chi-squared test for categorical variables and multiple linear regressions for continuous variables.

**Table 2:** Nutrient and meal intake of study participants based on the quartiles of nightly fasting duration in Korean adults.

	Men (n = 9683)					Women (n = 13,002)				
	Nightly Fasting Duration				*p* Value ^(1)^	Nightly Fasting Duration				*p* Value
	Q1 (Lowest)	Q2	Q3	Q4 (Highest)		Q1 (Lowest)	Q2	Q3	Q4 (Highest)	
	(n = 2680)	(n = 1954)	(n = 2728)	(n = 2321)		(n = 3524)	(n = 3073)	(n = 3627)	(n = 2778)	
Energy, kcal/day	2324 ± 18	2207 ± 22	2070 ± 18	1919 ± 21	<0.0001	1748 ± 13	1682 ± 12	1600 ± 12	1458 ± 14	<0.0001
Nutrient intake										
Energy from carbohydrates, %	61.8 ± 0.2	63.5 ± 0.3	63.3 ± 0.3	60.0 ± 0.3	<0.0001	64.4 ± 0.2	65.2 ± 0.3	64.4 ± 0.2	61.4 ± 0.3	<0.0001
Energy from protein, %	15.9 ± 0.1	15.4 ± 0.1	15.8 ± 0.1	16.5 ± 0.1	<0.0001	14.8 ± 0.1	14.7 ± 0.1	15.0 ± 0.1	15.3 ± 0.1	0.0001
Energy from fats, %	22.3 ± 0.2	21.1 ± 0.3	20.9 ± 0.2	23.6 ± 0.3	<0.0001	20.8 ± 0.2	20.1 ± 0.2	20.7 ± 0.2	23.3 ± 0.2	<0.0001
Dietary fiber, g/day	28.6 ± 0.3	28.8 ± 0.4	27.2 ± 0.3	22.3 ± 0.3	<0.0001	25.4 ± 0.3	24.7 ± 0.3	22.8 ± 0.3	18.7 ± 0.3	<0.0001
Meal intake										
Energy from main meals, %	77.5 ± 0.4	83.0 ± 0.4	85.6 ± 0.3	85.8 ± 0.3	<0.0001	75.9 ± 0.3	80.6 ± 0.4	82.6 ± 0.3	83.0 ± 0.3	<0.0001
Energy from breakfast, %	16.2 ± 0.3	18.4 ± 0.3	19.0 ± 0.3	11.2 ± 0.4	<0.0001	18.7 ± 0.3	20.4 ± 0.3	20.1 ± 0.3	13.0 ± 0.4	<0.0001
Energy from lunch, %	28.1 ± 0.3	30.4 ± 0.4	30.9 ± 0.4	33.4 ± 0.5	<0.0001	28.7 ± 0.3	30.2 ± 0.3	30.4 ± 0.3	34.0 ± 0.5	<0.0001
Energy from dinner, %	33.2 ± 0.4	34.1 ± 0.4	35.6 ± 0.4	41.1 ± 0.5	<0.0001	28.5 ± 0.3	29.9 ± 0.3	32.1 ± 0.3	36.0 ± 0.5	<0.0001
Energy from snacks, %	22.5 ± 0.4	17.0 ± 0.4	14.4 ± 0.3	14.2 ± 0.4	<0.0001	24.1 ± 0.3	19.4 ± 0.4	17.4 ± 0.3	17.0 ± 0.4	<0.0001

KNHANES, Korea National Health and Nutrition Examination Survey; Q, quartile. Data are presented as means ± standard errors (SEs) or numbers (weighted %). ^(1)^ *p* values were calculated using the chi-squared test for categorical variables and multiple linear regressions for continuous variables.

**Table 3:** Dietary behaviors of study participants based on quartiles of nightly fasting duration in Korean adults.

	Men (n = 9683)					Women (n = 13,002)				
	Nightly Fasting Duration				*p* Value ^(1)^	Nightly Fasting Duration				*p* Value
	Q1 (Lowest)	Q2	Q3	Q4 (Highest)		Q1 (Lowest)	Q2	Q3	Q4 (Highest)	
	(n = 2680)	(n = 1954)	(n = 2728)	(n = 2321)		(n = 3524)	(n = 3073)	(n = 3627)	(n = 2778)	
Dietary behaviors										
Eating episodes, times/d	6.3 ± 0.05	5.9 ± 0.05	5.1 ± 0.04	4.0 ± 0.04	<0.0001	6.2 ± 0.03	5.5 ± 0.03	4.9 ± 0.02	3.9 ± 0.03	<0.0001
Main meal episodes, times/d	2.7 ± 0.01	2.8 ± 0.01	2.7 ± 0.01	2.2 ± 0.01	<0.0001	2.7 ± 0.01	2.8 ± 0.01	2.7 ± 0.01	2.2 ± 0.01	<0.0001
Snack episodes, times/d	3.6 ± 0.05	3.1 ± 0.04	2.4 ± 0.04	1.7 ± 0.03	<0.0001	3.4 ± 0.03	2.8 ± 0.03	2.3 ± 0.03	1.7 ± 0.03	<0.0001
Nightly fasting duration, h	8.1 ± 0.1	11.1 ± 0.01	12.5 ± 0.01	15.6 ± 0.1	<0.0001	9.4 ± 0.04	11.9 ± 0.01	13.4 ± 0.01	16.1 ± 0.04	<0.0001
Time of first eating episode, h	5.5 ± 0.1	7.6 ± 0.03	8.4 ± 0.04	10.8 ± 0.1	<0.0001	6.6 ± 0.05	8.0 ± 0.03	8.8 ± 0.03	10.8 ± 0.05	<0.0001
Time of last eating episode, h	21.4 ± 0.03	20.5 ± 0.04	19.8 ± 0.04	19.2 ± 0.05	<0.0001	21.2 ± 0.03	20.1 ± 0.03	19.4 ± 0.03	18.7 ± 0.04	<0.0001
Energy in the morning (05:00 to 9:00 a.m.), %	15.7 ± 0.3	18.0 ± 0.4	17.4 ± 0.4	7.2 ± 0.3	<0.0001	18.9 ± 0.3	19.2 ± 0.3	16.9 ± 0.3	6.5 ± 0.3	<0.0001
Energy in the evening (06:00 to 9:00 p.m.), %	30.5 ± 0.4	34.5 ± 0.5	35.3 ± 0.4	37.1 ± 0.6	<0.0001	28.3 ± 0.4	31.3 ± 0.4	31.2 ± 0.4	31.8 ± 0.6	<0.0001
Energy in the night (after 9:00 p.m.), %	14.9 ± 0.4	5.2 ± 0.3	3.8 ± 0.3	3.9 ± 0.3	<0.0001	9.0 ± 0.3	3.2 ± 0.2	2.6 ± 0.2	2.0 ± 0.2	<0.0001
Energy from foods consumed outside the home, %	50.3 ± 0.8	42.4 ± 0.9	38.9 ± 0.8	39.0 ± 0.9	<0.0001	34.7 ± 0.6	30.7 ± 0.7	29.1 ± 0.7	33.0 ± 0.9	<0.0001

KNHANES, Korea National Health and Nutrition Examination Survey; Q, quartile. Data are presented as means ± standard errors (SEs) or numbers (%). ^(1)^ *p* values were calculated using the chi-squared test for categorical variables and multiple linear regressions for continuous variables.

**Table 4:** Adjusted odds ratios and 95% confidence intervals of type 2 diabetes mellitus based on quartiles, median, and continuous types of nightly fasting duration in Korean adults.

	Nightly Fasting Duration						
	Quartiles				*P* for trend ^(3)^	≥12.0 (M)/12.7 (W) vs. <12.0 (M)/12.7 (W)	Per 1 h Increase
	Q1 (Lowest)	Q2	Q3	Q4 (Highest)			
Men (n = 9683)	2680	1954	2728	2321		9683	9683
Cases (n)	419	372	531	312		1634	1634
Age-adjusted model ^(1)^	1.00	0.95 (0.78–1.15) ^(2)^	0.92 (0.77–1.09)	0.77 (0.63–0.94)	0.29	0.87 (0.76–0.99)	0.98 (0.95–1.00)
Multivariable-adjusted model	1.00	0.98 (0.81–1.19)	0.94 (0.79–1.13)	0.75 (0.61–0.91)	0.06	0.88 (0.77–1.01)	0.98 (0.95–1.00)
Women (n = 13,002)	3524	3073	3627	2778		13,002	13,002
Cases (n)	423	413	522	273		1631	1631
Age-adjusted model	1.00	0.92 (0.77–1.11)	0.93 (0.78–1.10)	0.86 (0.71–1.05)	0.29	0.94 (0.82–1.07)	0.98 (0.95–1.01)
Multivariable-adjusted model	1.00	0.92 (0.76–1.12)	0.90 (0.75–1.07)	0.77 (0.62–0.95)	0.43	0.89 (0.77–1.02)	0.97 (0.94–0.99)

KNHANES, Korea National Health and Nutrition Examination Survey; Q, quartile; OR, odds ratio; CI, confidence interval; M, men; W, women. ^(1)^ Age-adjusted model was adjusted for age (years, continuous); Multivariable-adjusted model was adjusted for age (years, continuous), educational level (elementary school, middle school, high school, or college), household income level (lower-middle, middle, upper-middle, or high), household type (single-person or multi-person), alcohol consumption (none, moderate, or high), smoking status (never, past, or current), regular physical activity (yes or no), and body mass index (kg/m^2^, continuous). ^(2)^ Adjusted odds ratio (95% confidence interval). ^(3)^ The *p* value for the trend was calculated using the median value of the nightly fasting duration in each quartile, treating it as a continuous variable in the model.

**Table 5:** Adjusted odds ratios and 95% confidence intervals of type 2 diabetes mellitus based on meal timing in Korean adults.

	Men (n = 9683)	Women (n = 13,002)
Cases (n)	1634	1631
First mealtime		
<09:00 a.m. (n = 14,788)	1.00	1.00
≥09:00 a.m. (n = 7897)	0.91 (0.77–1.08) ^(1)^	0.96 (0.81–1.14)
Last mealtime		
<09:00 p.m. (n = 14,853)	1.00	1.00
≥09:00 p.m. (n = 7832)	1.18 (1.02–1.37)	1.20 (1.02–1.40)
Energy in the morning (05:00 to 9:00 a.m.)		
None (n = 6410)	1.00	1.00
<25% (n = 8698)	1.09 (0.90–1.32)	0.93 (0.75–1.15)
≥25% (n = 7577)	1.09 (0.88–1.35)	1.10 (0.90–1.35)
Energy in the evening (06:00 to 9:00 p.m.)		
None (n = 2563)	1.00	1.00
<40% (n = 13,027)	1.35 (1.06–1.71)	1.38 (1.12–1.71)
≥40% (n = 7095)	1.40 (1.08–1.83)	1.32 (1.02–1.70)
Energy at night (after 9:00 p.m.)		
None (n = 17,038)	1.00	1.00
<25% (n = 4012)	1.14 (0.95–1.37)	1.14 (0.93–1.40)
≥25% (n = 1635)	0.89 (0.68–1.18)	1.61 (1.13–2.30)

KNHANES, Korea National Health and Nutrition Examination Survey; OR, odds ratio; CI, confidence interval. ^(1)^ Adjusted odds ratio (95% confidence interval). The multivariable-adjusted model was adjusted for age (years, continuous), educational level (elementary school, middle school, high school, or college), household income level (lower middle, middle, upper middle, or highest), household type (single-person or multi-person), alcohol consumption (none, moderate, or high), smoking status (never, past, or current), regular physical activity (yes or no), and body mass index (kg/m^2^, continuous).

## Data Availability

The datasets used and/or analyzed during the current study are available from the Korea National Health and Nutrition Examination Survey (KNHANES) official website at https://knhanes.kdca.go.kr/knhanes/sub03/sub03_02_05.do (accessed on 3 July 2022).
